# Integrating Optimal Heterogeneous Sensor Deployment and Operation Strategies for Dynamic Origin-Destination Demand Estimation

**DOI:** 10.3390/s17081767

**Published:** 2017-08-02

**Authors:** Senlai Zhu, Yuntao Guo, Jingxu Chen, Dawei Li, Lin Cheng

**Affiliations:** 1 School of Transportation, Nantong University, Se Yuan Road #9, Nantong 226019, China; zhusenlai@163.com; 2 Lyles School of Civil Engineering/NEXTRANS Center, Purdue University, 3000 Kent Avenue, West Lafayette, IN 47906, USA; 3 School of Transportation, Southeast University, Si Pai Lou #2, Nanjing 210096, China; chenjingxu@seu.edu.cn (J.C.); lidawei@seu.edu.cn (D.L.); gist@seu.edu.cn (L.C.)

**Keywords:** network sensor location problem, heterogeneous sensor deployment strategy, dynamic O-D demand estimation, heterogeneous sensor operation strategy

## Abstract

Most existing network sensor location problem (NSLP) models are designed to identify the number of sensors with fixed costs and installation locations, and sensors are assumed to be installed permanently. However, sometimes sensors are carried by individuals to collect traffic data measurements manually at fixed locations. Hence, their duration of operation for which traffic data measurements are collected is limited, and their costs are not fixed as they are correlated with the duration of operation. This paper proposes a NSLP model that integrates optimal heterogeneous sensor deployment and operation strategies for the dynamic O-D demand estimates under budget constraints. The deployment strategy consists of the numbers of link and node sensors and their installation locations. The operation strategy includes sensors’ start time and duration of operation, which has not been addressed in previous studies. An algorithm is developed to solve the proposed model. Numerical experiments performed on a network from a part of Chennai, India show that the proposed model can identify the optimal heterogeneous sensor deployment and operation strategies with the maximum dynamic O-D demand estimation accuracy.

## 1. Introduction

Traffic flows of transportation network can be characterized in various ways, such as origin–destination (O-D) demand, path flows, intersection turning movements, etc. These flows are essential inputs for traffic operational and management applications, such as traffic congestion control, route guidance, etc. However, it is difficult to estimate some types of flows such as O-D demand, while other types of flows can be observed due to recent advances in real-time sensing technologies, such as GPS, global system for mobile communications (GSM), Bluetooth, plate scanning, video camera, automatic vehicle identification (AVI), etc. With the traffic flow characteristics of data collected from these sensors, one can derive and analyze various traffic data measurements, including turning movements at intersections, traffic speed, density, and speed on lanes, and full or partial vehicle trajectories along the path. For example, a fixed camera or video can be used to collect flows information at an intersection. By processing the images or videos, turning movements in the intersection can be collected.

O-D demand has been estimated using traffic data measurements obtained through sensors with fixed locations and/or mobile sensors [1,2,3]. Sensors with fixed locations (e.g., video camera) can only be installed on certain locations (e.g., lanes and intersections), while mobile sensors (e.g., GPS) can move within the network. However, due to limited resources, sensors cannot be installed on every lane, intersection, and/or path in practice. Hence, a theoretical issue linked to O-D estimation problem is the identification of the optimal numbers of sensors and their installation locations in the network, that is, the network sensor location problem (NSLP). 

For sensors with fixed locations, existing NSLP models assumed by default that, once a sensor’s location is determined, it would be permanently installed at the location. This assumption may be true for some metropolitan regions with well-developed intelligence transportation systems. However, for some cities (especially for cities in developing countries, such as Nantong in Jiangsu Province, China), sensors are not allowed to be installed on the network permanently since the demand patterns and network topologies of these cities are constantly changing with the developing of cities. Instead, individuals equipped with sensors are assigned to different locations to collect traffic data measurements manually. In this case, each individual with a sensor can be treated as a “sensor”. These “sensors” cannot be installed permanently and the sensors’ duration of operation for which traffic data measurements are collected normally limited. The proposed study focuses on the NSLP for sensors with fixed locations and limited duration of operation.

In the literature, most existing NSLP models are designed for estimating static O-D demand, and traffic data measurements used in the models usually come from sensors located on links for detecting link flows only. Considering the inherent correlation between the O-D estimation and link count observations, several studies formulated NSLP as an O-D covering problem. For example, Lam and Lo [4] introduced a NSLP model to determine priorities for locating sensors using traffic flow volume and O-D coverage criteria. Yang et al. [5] used the maximum possible relative error (MPRE) criterion and the unknown true O-D demand to estimate the most possible deviation of the O-D demand. Yang and Zhou [6] further expanded the MPRE criterion by proposing four basic sensor location rules, namely the maximal flow fraction rule, the maximal flow interception rule, the O-D covering rule, and the link independence rule. Several of these rules were evaluated on a large-scale network by Yim and Lam [7]. Bianco et al. [8] introduced an iterative two-stage method to maximize the O-D demand coverage and reduce the MPRE value. Gan et al. [9] proposed a modified MPRE formulation with the expected relative error (the difference between the real and estimated O-D demands). Bierlaire [10] introduced the total demand scale (TDS) measure to quantify the difference between the minimum and maximum possible total demand estimates. Chen et al. [11] extended the TDS measure by generalizing it for different spatial levels. Several recent studies proposed NSLP models using statistical inference approaches. For example, Simonelli et al. [12] introduced a synthetic dispersion measure (SDM) based on the trace of the covariance matrix of the posterior demand estimates conditional upon existing sensor locations. Zhu et al. [13] utilized the SDM and proposed a stepwise algorithm to identify the optimal sensor deployment strategy for the static O-D demand and the unobserved link flows estimations.

Recent advances in real-time sensing technologies allow various types of traffic data measurements to be collected. For example, turning movements at intersections (nodes) can be measured by cameras or videos, and full or partial path flows can be obtained from GPS, AVI, plate scanning, etc. These advances also entail challenges such as handling various types of sensors in the NSLP, and many recent studies proposed different models to address these challenges for the static O-D estimation problem. Chen et al. [14] introduced a multi-objective model for AVI reader location strategy, and Chen et al. [15] extended the model to accommodate different travel demand patterns. Minguez et al. [16] and Castillo et al. [17] introduced different models to optimize the traffic plate scanning location strategy for O-D demand and route flow estimation under budget constraints. Yang et al. [18] and Chen et al. [19] proposed different methods to address the screen line-based traffic counting location problem. Hu et al. [20] introduced a bi-level optimization model to identify the optimal deployment strategy for license plate recognition and vehicle detector sensors.

Due to the time-varying characteristic, estimating dynamic O-D demand is substantially more complicated than the static O-D estimation. Hence, it is also challenging to design for the corresponding NSLP models. For the NSLP models with only link sensors, Eisenman et al. [21] introduced a Kalman filter conceptual framework to minimize the error in the dynamic O-D demand estimates in the NSLP. Fei et al. [22] further extended this framework using the TDS measure proposed by Bierlaire [8] to examine the NSLP under with and without budget constraints scenarios. Some studies also incorporated multiple types of sensor for the NSLP models in dynamic O-D demand estimation. For example, Asakura et al. [23] proposed an off-line least-squares model to simultaneously determine the identification rates of AVI data and the O-D demand. Zhou and List [24] introduced a model to maximize the expected information gain for the dynamic O-D demand estimation by locating a limited set of traffic counting stations and AVI readers in a network. Barcelo et al. [25] discussed the link covering and node covering formulations. Zhu et al. [26] proposed a model to determine the optimal heterogeneous sensor deployment strategy in terms of the numbers of link and node sensors and their installation locations for dynamic O-D estimation under a budget constraint. 

The NSLP has also been applied in other related domains, in addition to its use in the traditional O-D demand estimation problem, such as travel time estimation problem [26,27,28,29], network observability problem [30,31,32,33,34,35,36,37], path flow estimation [38,39], etc.

In the literature, existing NSLP models assumed sensors can be installed permanently on the network and the cost of sensor is fixed, the amount of information provided by a sensor depends only on its installation location. However, as mentioned before, individuals equipped with sensors are assigned on the field to collect traffic data measurements manually often, especially in developing countries due to fast city expansion and urbanization. These sensors’ duration of operation for which traffic data measurements are collected is normally limited. Hence, the cost of this type of sensor consists of both installation cost and operation cost, and the operation cost is normally correlated with a sensor’s duration of operation. One recent study by Zhu et al. [26] found that the amount of information provided by a sensor depends not only on its installation location, but also on its start time and duration of operation for which traffic data measurements are collected. They showed that the optimal sensor deployment strategy may change if the duration of operation for which traffic data measurements are collected is considered. Therefore, in addition, to identify the optimal heterogeneous sensor deployment strategy (sensor types, numbers of sensors, and installation locations), an ideal NSLP model should integrate heterogeneous sensor operation strategy (sensors’ start time and duration of operation for which traffic data measurements are collected) in the sensor deployment strategy for dynamic O-D estimation.

The objective of this study is to propose a NSLP model that integrates the optimal heterogeneous sensor deployment and operation strategies to maximize the quality of the dynamic O-D demand estimates under a budget constraint. Two types of sensors are considered in the proposed NSLP model, including link sensors (e.g., inductive loop detectors, magnetic detectors, etc.) and node sensors (e.g., camera or video), and these sensors are assumed to be operated by individuals equipped with sensors having limited duration of operation. In this study, link sensors are used to count vehicles on lanes or a set of lanes in the network to collect link flows, and node sensors are installed at intersections to detect turning movements. The sensor deployment strategy includes the numbers of link and node sensors and their installation locations, while the sensor operation strategy consists of sensors’ start time and duration of operation for which traffic data measurements are collected, which has not been addressed in previous studies. Sensors will be assigned at fixed locations within the analysis period, while their operation durations may vary. The quality of the dynamic O-D demand estimates is measured by the variability in the O-D demand estimation, and the trace of the covariance matrix of the posterior O-D demand estimates is used to measure the variability. An assumption was made that each time-dependent O-D demand is a random variable. We also assume that the cost of a node sensor is higher than a link sensor. To factor the correlation between operation cost of a sensor and its duration of operation for which traffic data measurements are collected, operation cost of a sensor is assumed to be positively linearly correlated with its duration of operation. To study the impact of start time and duration of operation for which traffic data measurements are collected on the optimal heterogeneous sensor deployment strategy, we compare the optimal heterogeneous sensor deployment and operation strategies with different unit sensor operation cost in the case study.

An integrated sensor location and operation algorithm that avoids matrix inversions is introduced to solve the proposed NSLP model. Since optimal sensor deployment strategy and operation strategy cannot be determined simultaneously due to the costs differences between link and node sensors, the proposed algorithm first fixes a sensor operation strategy (sensors’ start time and duration of operation for which traffic data measurements are collected), and with this operation strategy, the algorithm selects the sensor deployment strategy under a budget constraint with the lowest variability in the dynamic O-D demand estimates. This process is repeated for other sensor operation strategies with different sensors’ start time and duration of operation. It then compares the selected sensor deployment strategies for various sensor operation strategies, and identifies the optimal sensor deployment and operation strategies as the one with the lowest variability in the dynamic O-D demand estimates.

The remainder of the paper is organized as follows. The next section formulates the proposed NSLP model. Then, an integrated sensor location and operation algorithm is proposed for solving the NSLP model under the multivariate normal distribution assumption. Numerical experiments follow to demonstrate the performance of the proposed model and its solution algorithm. Finally, some conclusions are given.

## 2. Model Formulation

Firstly, the relationships among variables considered in the proposed NSLP model are analyzed. The considered variables include time-dependent O-D demand, path flows, link flows, and node turning movements. Then, based on these relationships, the proposed NSLP model is formulated. 

### 2.1. Relationships among Variables Considered in the NSLP Model

To formulate the proposed NSLP model, we assume linear relationships among the considered variables (time-dependent O-D demand, link flows, path flows, and node turning movements) by the assignment proportions:

(1) $$f_{i,k,t}=p_{i,k,t}d_{i,t}$$

(2) $$v_{j}=\underset{i}{\sum }\underset{k}{\sum }\underset{t}{\sum }\varphi_{i,k,t,j}\;f_{i,k,t}$$

(3) $$s_{j_{a,b}}=\underset{i}{\sum }\underset{k}{\sum }\underset{t}{\sum }\psi_{i,k,t,j_{a,b}}\;f_{i,k,t}$$

where $f_{i,k,t}$ is the number of users of O-D pair i choosing path k with a departure time of t, $p_{i,k,t}$ is the proportion of users of O-D pair i with a departure time of t choosing path *k*, and $d_{i,t}$ is the flow of O-D pair i with a departure time of t. The departure time of O-D pair or path means the time that traffic flow started from the origin node or the first node of the path, respectively. $v_{j}$ is the flow on link j during the considered time period (constrained by its start time and duration of operation for which traffic data measurements are collected), $\varphi_{i,k,t,j}$ is equal to the proportion of users of path k of O-D pair i with departure time t choosing link j with departure time $\tau_{j}$ included in the considered time period, and the departure time $\tau_{j}$ is the time that traffic flow started from node j, $s_{j_{a,b}}$ is the number of users traveling from upstream node a to downstream node b connected by node j during the considered time period, $\psi_{i,k,t,j_{a,b}}$ is equal to the proportion of users of path k of O-D pair i. with departure time t raveling from upstream node a. to downstream node b connected by node j with departure time $\tau_{j}$ included in the considered time period. $a\in N_{u}$ and $b\in N_{d}$, where $N_{u}$ represents the set of upstream nodes of node j and $N_{d}$ is the set of downstream nodes of node j. These assignment proportions $p_{i,k,t}$, $\varphi_{i,k,t,j}$ and $\psi_{i,k,t,j_{a,b}}$ can be obtained by solving the DUE problem.

To analysis in the matrix form, we define matrices D, $F_{i}$, and F as follows:

(4) $$D={\left[d_{1,1},d_{1,2},\ldots ,d_{1,t},\ldots ,\underset{\mathrm{time}-\mathrm{dependent}\;\mathrm{demand}\;\mathrm{of}\;O-D\;\mathrm{pair}\;i}{\underbrace{d_{i,1},d_{i,2},\ldots ,d_{i,t}\ldots }},\ldots \right]}^{T}$$

(5) $$F_{i}={\left[f_{i,1,1},f_{i,1,2},\ldots ,f_{i,1,t},\ldots ,\underset{\mathrm{time}-\mathrm{dependent}\;\mathrm{flow}\;\mathrm{of}\;\mathrm{path}\;k\;\mathrm{from}\;O-D\;\mathrm{pair}\;i}{\underbrace{f_{i,k,1},f_{i,k,2},\ldots ,f_{i,k,t}\ldots }},\ldots \right]}^{T}$$

(6) $$F={\left[F_{1}^{T},F_{2}^{T},\ldots ,F_{i}^{T},\ldots \right]}^{T}$$

(7) $$P={\left[P_{1,1}^{T},P_{1,2}^{T},\ldots ,P_{2,1}^{T},P_{2,2}^{T},\ldots ,P_{i,1}^{T},P_{i,2}^{T},\ldots \right]}^{T}$$

where D is the vector of all time-dependent O-D demands being considered, $F_{i}$ is the vector of all time-dependent path flows of O-D pair i, F is the vector of all time-dependent path flows, and P is the vector of all assignment proportions in Equation (1), in which $P_{i,t}$ is a m×s matrix (m is the total number of paths for O-D pair i with a departure time of t, and s is the dimension of D).

The (k, j)*^th^* element $P_{i,t}\left(k,j\right)$ of $P_{i,t}$ is set as:

(8) $$P_{i,t}\left(k,j\right)=\{\begin{array}{c} p_{i,k,t}\;\mathrm{if}\;j=\left(i-1\right)*\left|T\right|+t\\ 0\;\mathrm{otherwise}\; \end{array}$$

where |T| is the number of time intervals.

Then, the path flows satisfy the following flow conservation condition:

(9) F=PD

The relationship between O-D demand and path flows can be derived by Equation (9). However, path flows do not need to be collected in the proposed model, since the main purpose of introducing path flows in the model is to use them as intermediate variables to reflect the relationship among O-D demand, link flows and turning movements. 

Similarly, define matrices $\varphi_{i,j}$, $\varphi_{j}$, φ, V as follows:

(10) $$\varphi_{i,j}=\left[\varphi_{i,1,1,j},\varphi_{i,1,2,j},\ldots ,\varphi_{i,k,1,j},\varphi_{i,k,2,j}\ldots \right]$$

(11) $$\varphi_{j}=\left[\varphi_{1,j},\varphi_{2,j},\ldots ,\varphi_{i,j},\ldots \right]$$

(12) $$\varphi ={\left[\varphi_{1}^{T},\varphi_{1}^{T},\ldots ,\varphi_{j}^{T}\ldots \right]}^{T}$$

(13) $$V={\left[v_{1},v_{2},\ldots ,v_{j},\ldots \right]}^{T}$$

(14) $$\psi_{i,j}=\left[\psi_{i,j}{}_{i,1,1,j},\psi_{i,j}{}_{i,1,2,j},\ldots ,\psi_{i,j}{}_{i,k,1,j},\psi_{i,j}{}_{i,k,2,j},\ldots \psi_{i,j}{}_{i,k,t,j}\ldots ,\ldots \right]$$

(15) $$\psi_{j}=\left[\psi_{1,j},\psi_{2,j},\ldots ,\psi_{i,j},\ldots \right]$$

(16) $$\psi ={\left[\psi_{1}^{T},\psi_{2}^{T},\ldots ,\psi_{j}^{T},\ldots \right]}^{T}$$

(17) $$S={\left[S_{1},S_{2},\ldots ,S_{j},\ldots \right]}^{T}$$

where V is the vector of all considered link flows, and φ is the corresponding assignment proportion vector. $\varphi_{i,j}$ is the vector of proportion s for each time-dependent path of O-D pair i that chooses link j. $\varphi_{j}$ is the vector of proportions related to each time-dependent path for each O-D pair that chooses link j. S is the vector of all considered turning movements, and Ψ is the corresponding assignment proportion vector. $S_{j}$ is the set of all the turning movements at node j, and $\psi_{i,k,t,j}$ is the vector of proportions related to path k of O-D pair i with departure time t choosing each turning movement at node j. $\Psi_{i,j}$ is the vector of proportions related to each time-dependent path of O-D pair i choosing each turning movement at node j. $\Psi_{j}$ is the vector of proportions related to each time-dependent path for each O-D pair choosing each turning movement at node j.

By considering error terms ε and η, the following linear relationship can be defined:

(18) V=φF+ε=φPD+ε

(19) S=ψF+η=ψPD+η

where $\varepsilon =\left(\varepsilon_{1},\varepsilon_{2},\ldots \right)$ and $\eta =\left(\eta_{1},\eta_{2},\ldots \right)$ are mutually independent random variables with zero mean.

In summary, according to Equations (9), (18) and (19), the random variables in the proposed model can be expressed through the following linear relationships:

(20) $$\left(\begin{array}{c} D\\ F\\ V\\ S \end{array}\right)=\left(\begin{array}{ccc} I & 0 & 0\\ P & 0 & 0\\ \varphi P & I & 0\\ \Psi P & 0 & I \end{array}\right)\left(\begin{array}{c} D\\ \varepsilon \\ \eta \end{array}\right)$$

In Equation (20), all the proportion vectors (P, φ and Ψ) can be obtained by solving the dynamic equilibrium problem (DUE) based on a prior O-D demand. 

The traffic demands of all time-dependent O-D pairs are assumed to follow multivariate normal (MVN) distributions in this study, and similar assumptions have been made in previous studies [40,41]. This assumption is reasonable because these random variables are the outcome of a large number of independent Bernoulli experiments in which the users decide their destinations and routes. 

Notably, based on Equation (20), if D is multivariate normal distributed with mean E(D) and variance $\Sigma_{D}$, ε is multivariate normal distributed with mean E(ε) and variance $\Sigma_{\varepsilon }$, and η is multivariate normal distributed with mean E(η) and variance $\Sigma_{\eta }$, the prior variance-covariance matrix $\Sigma_{\left(D,F,V,S\right)}$ of all considered variables is:

(21) $$\Sigma_{\left(D,F,V,S\right)}=\left(\begin{array}{cccc} \Sigma_{D} & \Sigma_{D}P^{T} & \Sigma_{D}{\left(\varphi P\right)}^{T} & \Sigma_{D}{\left(\Psi P\right)}^{T}\\ P\Sigma_{D} & P\Sigma_{D}P^{T} & P\Sigma_{D}{\left(\varphi P\right)}^{T} & P\Sigma_{D}{\left(\Psi P\right)}^{T}\\ \varphi P\Sigma_{D} & \varphi P\Sigma_{D}P^{T} & \varphi P\Sigma_{D}{\left(\varphi P\right)}^{T}+\Sigma_{\varepsilon } & \varphi P\Sigma_{D}{\left(\Psi P\right)}^{T}\\ \Psi P\Sigma_{D} & \Psi P\Sigma_{D}P^{T} & \Psi P\Sigma_{D}{\left(\varphi P\right)}^{T} & \Psi P\Sigma_{D}{\left(\Psi P\right)}^{T}+\Sigma_{\eta } \end{array}\right)$$

Based on the variance-covariance matrix, the relationships among the variables being considered can be derived.

As we have obtained the prior variance-covariance matrix of all the variables, the covariance matrix of all the variables can be updated based on some observed variables using the following equations [42,43]:

(22) $$\Sigma_{XY|Z=z}=\Sigma_{XY}-\Sigma_{XZ}\Sigma_{ZZ}^{-1}\Sigma_{YZ}$$

where X and Y are both the components of (D,F,V,S); $\Sigma_{ZZ}$ is the covariance matrix of the observation Z; $\Sigma_{XZ}$ is the covariance matrix of X and Z; $\Sigma_{YZ}$ is the covariance matrix of Y and Z; $\Sigma_{XY}$ is the covariance matrix of Y and X; and $\Sigma_{XY|Z=z}$ is the posterior covariance matrix of Y and X. 

### 2.2. Formulation of the NSLP Model

To calculate the variance-covariance matrix of all considered variables in Equation (21), we need to know the prior distribution $\phi_{D}$ of O-D demand (i.e., E(D) and $\Sigma_{D}$ in Equation (21)). The prior distribution of O-D demand can be obtained from many different sources, including simulation, historical or real data measurements. Define Z as a sensor deployment strategy with known counted flows as Z=z (including observed node turning movements and link flows). Then, by updating the counted flows, $\phi_{D|Z=z}$, is obtained, which presents the posterior distribution of the time-dependent O-D demand. Hence, the proposed model is capable of incorporating the prior and posterior variability of the dynamic O-D demand.

O-D estimation variability cannot be measured directly, so some indirect quantitative measurements are used. Variance of a single O-D demand can represent the variability of this single O-D demand. The trace of the covariance matrix of O-D demand equals to the sum of variances of all O-D demand estimates. In this study, we adopt the trace $Tr\left(\Sigma_{D|Z=z}\right)$ to represent the variability of the time-dependent O-D demand conditional on the counted flows Z=z, similar to approaches used in past studies [12,13]. Then, the variability of the posterior random vector D|Z=z is defined as the average of $Tr\left(\Sigma_{D|Z=z}\right)$, as follows:

(23) $$E\left[Tr\left(\Sigma_{D|Z=z}\right)\right]=\int_{\Omega_{Z}}Tr\left(\Sigma_{D|Z=z}\right)\phi_{Z}\partial z.$$

where $\phi_{Z}$ is the density function of the random variable Z, and $\Omega_{Z}$ is its domain.

Under the assumption of multivariate normal distribution, it can be shown from Equation (22) that the conditional variance $\Sigma_{XY|Z=z}$ does not depend on Z (i.e., z). Thus, it can lead to a significant simplification to calculate the posterior variability of the posterior random vector D|Z=z as introduced in the next section.

According to Equation (23), under the assumption of multivariate normal distribution, the posterior variability of the posterior random vector D|Z=z can be rewritten as:

(24) $$E\left[Tr\left(\Sigma_{D|Z=z}\right)\right]=Tr\left(\Sigma_{D}-\Sigma_{DZ}\Sigma_{ZZ}^{-1}\Sigma_{DZ}\right)$$

Hence, the NSLP can be formulated as the problem of identifying the optimal heterogeneous sensor deployment strategy $Z^{*}$, which has the minimum variability of the posterior random vector D|Z=z in the domain $\Omega_{Z}$, subject to constraints related to budget, start time and duration of operation for which traffic data measurements are collected. Then, the proposed model for dynamic O-D demand estimation is formulated as follow:

(25) $$\min Tr\left(\Sigma_{D}-\Sigma_{DZ}\Sigma_{ZZ}^{-1}\Sigma_{DZ}\right)$$

(26) $$s.t.\;b_{v}L+b_{s}S\leq b_{max}$$

(27) $$b_{v}=c_{v}+e_{v}\tau$$

(28) $$b_{s}=c_{s}+e_{s}\tau$$

(29) $$t_{st}\;\in T_{st}$$

where $b_{v}$ is the cost of a link sensor, $b_{s}$ is the cost of a node sensor, $b_{max}$ is the available budget, L is the cardinality of the identified link set, and S is the cardinality of the identified node set, $c_{v}$ is the fixed installation cost of a link sensor, which does not vary with the time, $e_{v}$ is the operation cost of a link sensor per unit of time, $c_{s}$ is the fixed installation cost of a node sensor, $e_{s}$ is the operation cost of a node sensor per unit of time, τ is duration of operation for which traffic data measurements are collected, $t_{st}$ is the start time for which traffic data measurements are collected, $T_{st}$ is the set of all considered start time.

In the proposed NSLP model, Equation (26) is the budget constraint. Equations (27) and (28) are cost functions for a link sensor and a node sensor, respectively. In practice, sometimes the costs for a link sensor and a node sensor are normally related to sensors’ duration of operation. To factor this, based on Equations (27) and (28), operation costs of a link sensor or a node sensor are assumed to be positively linearly correlated with their duration of operation. Without loss of generality, other types of cost function are also applicable in the proposed model, such as exponential, quadratic forms etc. For demonstration purpose, we use linear functions in this paper. Equation (29) is the start time constraint. Since assignment proportion vectors P, φ and Ψ in Equation (21) vary with the start time and duration of operation for which traffic data measurements are collected, solving the proposed NSLP model can provide not only the optimal heterogeneous sensor deployment strategy, but also the optimal operation strategy, including start time and duration of operation for which traffic data measurements are collected. In summary, sensors’ operation duration is constrained by Equation (29). Once the duration is determined, the number of sensors in Z is constrained by Equations (26)–(28), and sensors’ locations are constrained by the objective function Equation (25).

The resulting sensor deployment and operation strategies depend on the variability of both prior and Bayesian posterior traffic flow estimates. The posterior traffic flow estimates are related to the correlation of link flows, node turning movements and OD flows, which depends on the network structure and the users’ travel behavior. In summary, the objective function incorporates the variability of prior traffic flow estimates, the network structure, the users’ travel behavior and the sensor deployment and operation strategies.

## 3. Algorithm

To solve the NSLP model, the optimal operation strategy and the corresponding optimal sensor deployment strategy cannot be determined simultaneously. Therefore, the proposed integrated sensor location and operation algorithm first fixes operation strategy (sensors’ start time and duration of operation for which traffic data measurements are collected). It means the costs for a link sensor and a node sensor are fixed once the operation strategy is fixed. Then, the proposed algorithm determines the corresponding sensor deployment strategy with the lowest variability in the dynamic O-D demand estimates. The proposed algorithm has the following steps:

*Step 0*: Initialization: Define the initial and maximum values of start time $t_{st}$ for which traffic data measurements are collected to be $t_{st}^{0}$ and $t_{st}^{max}$, respectively. Define the minimum and maximum values of duration of operation τ for which traffic data measurements are collected to be $\tau_{min}$ and $\tau_{max}$, respectively. Define $\hat{Z}$ as the chosen optimal heterogeneous sensor deployment strategy, and Z as the current optimal heterogeneous sensor deployment strategy. The initial values of $\hat{Z}$ and Z are both set to be null sets. Define $\lambda^{min}$ as the minimum value of the objective function, whose initial value is $\lambda^{min}=tr\left(\Sigma_{D}\right)$. Define $\hat{t_{st}}$ and $\hat{\tau }$ as the chosen optimal start time and duration of operation for which traffic data measurements are collected, whose initial values are set to be $t_{st}^{0}$ and $\tau_{min}$, respectively.

*Step 1*: Sensor cost calculation: Based on τ, calculate the costs for a link sensor and a node sensor ($b_{v}$ and $b_{s}$ in Equations (27) and (28), respectively).

*Step 2*: Deployment strategy optimization with fixed operation strategy: Determine the heterogeneous sensor deployment strategy with the lowest variability in the dynamic O-D demand estimates under fixed $b_{l}$, $b_{s}$, τ and $t_{st}$.

*Step 2.1*: Solve the DUE problem to acquire the choice proportion matrices P, φ and Ψ.

*Step 2.2*: Use Equation (20) to calculate the prior variance and covariance of all considered variables.

*Step 2.3*: Define the maximum number of node sensors $n^{max}$ under the predetermined budget constraint $b_{max}$, and $n^{max}=int\left(b_{max}/b_{s}\right)$. Define m as the number of node sensors, with an initial value set as 0.

*Step 2.4*: Calculate the maximum number of link sensors $l^{max}$ ($l^{max}=int\left[\left(b_{max}-b_{s}m\right)/b_{v}\right)]$) under the budge constraint. l is the number of the identified link sensor locations, and n is the number of the identified node sensor locations. The initial values of l and n are 0. μ is the current value of the objective function, with an initial value of $\mu =tr\left(\Sigma_{D}\right)$.

*Step 2.5*: Define link or node $Z^{*}$ as the next sensor location that minimizes the objective function, where $Z^{*}$ can be calculated as follows:

(30) $$Z^{*}=\arg\;\min Tr\left(\Sigma_{D}-\Sigma_{DZ}\Sigma_{ZZ}^{-1}\Sigma_{DZ}\right)$$

If n<m and $l<l^{max}$, the newly identified sensor can be a link sensor or a node sensor; if n<m and $l=l^{max}$, the newly identified sensor can only be a node sensor; and if n=m and $l<l^{max}$, the newly identified sensor can only be a link sensor.

*Step 2.6*: Update the covariance, variance, and μ of the objective function. The covariance and variance of traffic flows can be updated as follows:

(31) $$\Sigma_{XY|Z^{*}}=\Sigma_{XY}-\Sigma_{XZ^{*}}\Sigma_{YZ^{*}}/\sigma_{Z^{*}Z^{*}}$$

where X and Y are the components of (D,F,V,S), $Z^{*}$ is the newly identified sensor location, and $\sigma_{Z^{*}Z^{*}}$ (a scalar) is the variance of $Z^{*}$.

*Step 2.7*: If the newly identified sensor is a link sensor, set l=l+1. If not set n=n+1. If $l=l^{max}$ and n=m, go to *Step 2.8*. Otherwise, go back to *Step 2.5*.

*Step 2.8*: Identify the optimal sensor deployment strategy Z with the defined number of node sensors as m. If $\mu <\lambda^{min}$, set $\lambda^{min}=\mu$, $\hat{Z}=Z$, $\hat{t_{st}}=\;t_{st}$, and $\;\hat{\tau }=\tau$.

*Step 2.9*: If $m<n^{max}$, set m=m+1 and initialize the variance and covariance of all variables (using the values acquired by *Step 2.2*). Set Z as a null set, and go back to *Step 2.4.* If not, go to *Step 3.*

*Step 3*: Operation strategy adjustment: If $t_{st}<t_{st}^{max}$ and $\tau <\;\tau_{max}$, add duration of operation $\tau =\;\tau +\tau^{interval}$, go to *Step 1*. If $t_{st}<t_{st}^{max}$ and $\tau ={\;\tau }_{max}$, add start time $t_{st}=\;t_{st}+t_{st}^{interval}$ and set $\tau ={\;\tau }_{min}$, and go to *Step 1*. If $t_{st}=t_{st}^{max}$ and $\tau ={\;\tau }_{max}$, stop the algorithmic process. Specify the optimal heterogeneous sensor deployment strategy $\hat{Z}$, and the optimal heterogeneous sensor operation strategy, including the optimal start time $\hat{t_{st}}$ and duration of operation $\hat{\tau }$ for which traffic data measurements are collected.

This algorithm allows us to sequentially change operation strategies (sensors’ start time and duration of operation for which traffic data measurements are collected), and, for each operation strategy, a corresponding sensor deployment strategy with the lowest variability is selected. Then, we compared the selected sensor deployment strategy for different operation strategies, and determine the best sensor operation and deployment strategies with the lowest variability. In *Step 3*, $t_{st}^{interval}$ and $\tau^{interval}$ are time intervals between considered start time and duration of operation, respectively. The values of $t_{st}^{interval}$ and $\tau^{interval}$ can be determined by operators.

*Step 2* is to determine the sensor deployment strategy with the lowest variability under a fixed operation strategy. In this step, the optimal numbers of link and node sensors cannot be determined simultaneously due to their cost difference. Hence, the proposed algorithm first specifies a given number of node sensors in the network under a budget constraint, and identifies the sensor deployment strategy with the lowest variability in the dynamic O-D demand estimates. Then, the proposed algorithm compares the identified sensor deployment strategy for different numbers of node sensors, and determines the sensor deployment strategy under the fixed operation strategy as the one with the lowest variability. In summary, all the possibilities have been compared and the best one is selected in *Step 2.8.*

The most computationally demanding step in the aforementioned algorithm is calculating the inverse of $\Sigma_{ZZ}$ (i.e., $\Sigma_{ZZ}^{-1}$). Interestingly, if we sequentially update one observed variable at a time in Equation (21), it avoids matrix inverse calculation, because $\Sigma_{DZ}$ is a column vector and $\Sigma_{ZZ}$ is a scalar (i.e., $\Sigma_{ZZ}=\sigma_{\mathrm{ZZ}}$). In *Step 2.5*, if the total number of considered links and turning movements is a and the number of identified sensor locations is *c*, the number of calculations needed is linearly correlated with the number of the remaining links and turning movements, which is the value of a−c. As most of the computational efforts are related to *Step 2.5*, the computational time of solving the proposed NSLP model is linearly correlated with the number of links and turning movements considered in the network. In some cases, more than one sensor location may be identified by the proposed algorithm in *Step 2.5* if these locations can provide the same covariance matrix reduction of the dynamic O-D demand estimates, which could entail additional computational efforts. The proposed algorithm is unable to determine which one of them should be included in the optimal sensor deployment and operation strategies. In this case, it needs to first identify all deployment and operation strategies with the same covariance matrix reduction of O-D demand estimates, and then choose the best one with the lowest variability. 

In addition, *Step 2* can also determine the priorities of the identified sensor locations. It means that the sensor location that is more important and effective in terms of improving O-D demand estimates will be identified preferentially by the proposed algorithm. In real-world application, the priorities can be used to determine which locations and the corresponding sensor types are more important and useful. The proposed algorithm can also be applied to solve the NSLP model in networks with existing sensors, by allocating these sensors in *Step 0*.

## 4. Numerical Experiments

**Figure 1:**
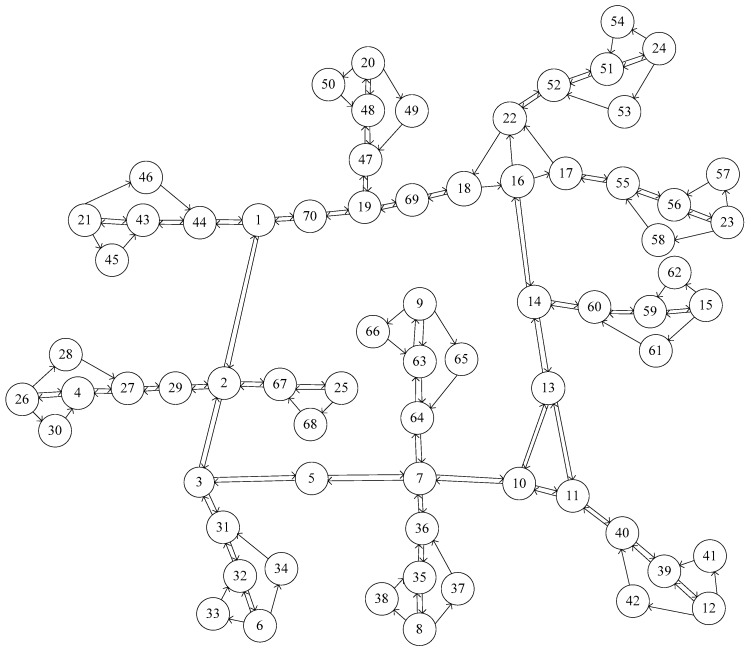
Network from a part of Chennai.

In this section, we evaluate the performance of the proposed NSLP models and algorithm using a network from a part of Chennai, India (see Figure 1). It includes 11 traffic analysis zones (100 O-D pairs), 70 nodes, and 141 directed links. The O-D matrix is time-dependent with eight 15-min intervals. The analysis period is two hours (from 8:00 a.m. to 10:00 a.m.). The time-dependent O-D matrix has 800 records (8 time intervals for 100 O-D pairs). The historical available for the network are used to derive the prior O-D demand. The budget is set as 400.

The cost functions of link and node sensors in Equations (27) and (28) are set as Equations (32) and (33), respectively:

(32) $$b_{v}=10+15\tau .$$

(33) $$b_{s}=20+30\tau$$

where the unit of time (i.e., t) is set to be 30 min. The numerical experiments were conducted using Python 2.7 and DYNASMART-P 1.3.0, and the prior link and node traffic data measurements are obtained by solving the dynamic user equilibrium problem using the prior O-D demand. A time-dependent shortest path (TDSP) method is used to solve the DUE paths in DYNASMART in each iteration. The method of successive averages is used to generate TDSPs for each departure time. The proposed algorithm is coded using Python 2.7. The computer system used to conduct the experiments is equipped with an Intel Core i7-4800MQ 2.70 GHz CPU and 8 GB RAM.

**Figure 2:**
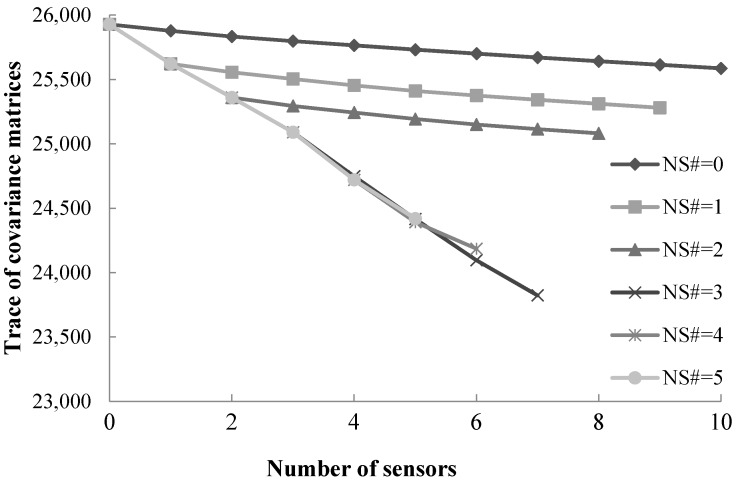
Traces of covariance matrices of dynamic O-D demand estimation after updating each identified sensor location with different given number of node sensors and a fixed sensor operation strategy.

To demonstrate the impact of integrating the operation strategy in the NSLP on the optimal sensor deployment strategy, numerical experiments with a fixed operation strategy are performed. Figure 2 shows the plots of the traces of the covariance matrices of the dynamic O-D demand estimation for the proposed NSLP model with fixed operation strategy (fixed start time and duration of operation for which traffic data measurements are collected). NS# represents the number of allowed node sensors in the network. Only *Step 2* of the proposed algorithm is needed. The start time is set to be 8:30 a.m., and the duration of operation for which traffic data measurements are collected is set to be one hour, from 8:30 a.m. to 9:30 a.m. The proposed algorithm can prioritize the deployed sensor by the amount of information provided during its operation time. Node sensors are identified by the algorithm first in each case in Figure 2. For example, when NS# = 3, the first three sensor locations are node sensor locations. This is because a node sensor can provide a greater amount of updated information compared to a link sensor. A node sensor can detect several turning movements (e.g., 12 turning movements at a traditional four-way intersection), and a link sensor can only detect one link. Each turning movement can be treated as a sub-path, and can provide more detailed information about traffic flows. A larger amount of updated information implies that more information can be collected from a node sensor to update the variance-covariance matrix, which can reduce the variability of the dynamic O-D demand estimations, compared to that from a link sensor. Thus, node sensors are normally identified preferentially by the proposed algorithm.

As shown in Figure 2, the traces decrease as each sensor is added. More sensors imply that more information can be collected, which results in the reduction of the variability of the dynamic O-D demand estimates. When a node sensor is added, the traces decrease more rapidly compared to a link sensor. This is because a node sensor can provide a greater amount of updated information than a link sensor. However, this does not imply that more node sensors can guarantee a lower variability of the O-D demand estimates under a budge constraint. As shown in Figure 2, the optimal heterogeneous sensor deployment strategy has only three node sensors, while the maximum number of allowed node sensors is 6. Despite a node sensor provides a greater amount of updated information than a link sensor, a link sensor is much cheaper than a node sensor. More node sensors can imply fewer link sensors, leading to a tradeoff in terms of the desirable numbers of each sensor type under a budget constraint. In summary, the proposed algorithm can be used to determine the optimal numbers for both the link sensors and node sensors with a fixed operation strategy under a budget constraint.

**Figure 3:**
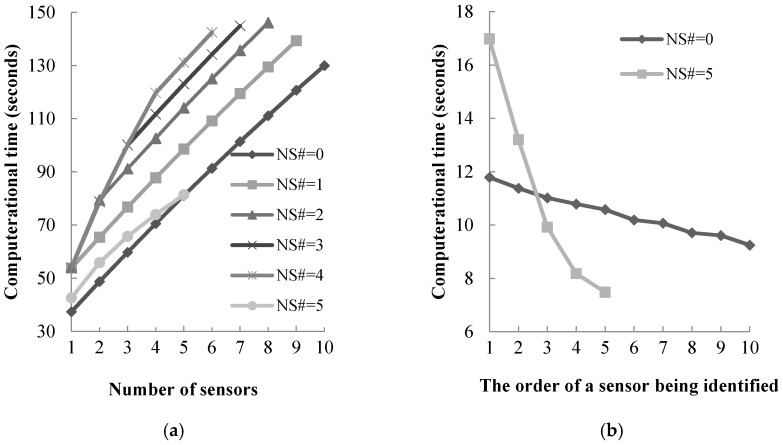
Computational time using the proposed algorithm: (**a**) cumulative computational time for each given number of node sensors; and (**b**) computational time for identifying each sensor.

Figure 3 shows the computational time to solve the proposed NSLP model using the proposed algorithm with a fixed operation strategy. Figure 3a shows the cumulative computational time for each given number of node sensors. Figure 3b illustrates the computational time for identifying each sensor in the scenario with only link sensors (NS# = 0) with that for the scenario with only node sensors (NS# = 5).

As shown in Figure 3a, with only link sensors (NS# = 0) or node sensors (NS# = 5) included, the cumulative computational times are approximately positively linearly correlated with the number of identified link sensor locations or node sensor locations. The linear correlation between the cumulative computational time and the number of identified link sensor locations or node sensor locations implies that when more sensors being installed in the network as the budget and size of the network increase, the cumulative computational time will not increase significantly. This facilitates the application of the proposed NSLP model and algorithm in real world with larger networks. Figure 3a also shows that the computational time to identify a node sensor’s location is normally larger than that to identify a link sensor’s location, with NS# = 2, NS# = 3 and NS# = 4. This is because a node sensor can provide a greater amount of updated information compared to a link sensor, so more computational time is needed to update the information in Equations (30) and (31). 

The proposed algorithm can also determine the priorities of the identified sensor locations. It means that the sensor location that is more important and effective in improving the dynamic O-D demand estimates will be identified preferentially by the proposed algorithm. As illustrated in Figure 3b, sensor locations with higher priorities cost much more computational time to be identified compared to the ones with lower priorities. As discussed in previous section, most of the computation burden in the proposed algorithm is in *Step 2.5*, and the computational time of *Step 2.5* is linearly correlated with the number of remaining links and turning movements (those unidentified locations) in the network. When the priority of a sensor location is lower, the number of unidentified links and turning movements is smaller, because other locations with higher priorities have been identified. Hence, the computational time to identify sensor locations with lower priorities is shorter than that of sensor locations with high priorities.

Table 1 shows the heterogeneous sensor deployment strategies with the lowest variability in the dynamic O-D demand estimates for different sensor operation strategies. In terms of the notation in Table 1, for example, 2(n) represents a node sensor located at node 2, while 10–13 represents a link sensor on link 10–13 with upstream node 10 and downstream node 13.

It can be seen from Table 1 that the sensor deployment strategies with the lowest variability under different sensor operation strategies are different. The amount of updated information provided by each sensor are different for different time intervals since their corresponding O-D demands and users’ travel behaviors are different, which illustrates the time-varying characteristics of the amount of updated information provided by each sensor. Hence, if the sensor operation strategy changes, its corresponding sensor deployment strategy with the lowest variability also changes.

As shown in Table 1, the optimal sensor deployment and operation strategies obtained have an operation strategy with a start time of 8:30 a.m. and a duration of operation for which traffic data measurements are collected of one hour. For a sensor, a longer duration of operation can potentially provide a greater amount of updated information, which leads to lower variability of the O-D demand estimates. However, our results show that a longer duration of operation does not necessarily lead to a lower variability of dynamic O-D demand estimates. As shown in Table 1, when the start time are 8:30 a.m., 9:00 a.m., 9:30 a.m. and 10:30 a.m., the corresponding duration of operation for sensor deployment strategy with the lowest variability are 60 min, 30 min, 60 min, and 90 min, respectively. In addition, more sensors in the network do not necessarily lead to lower variability of dynamic O-D demand estimates. As illustrated in Table 1, the optimal number of sensors for start time 8:30 a.m., 9:30 a.m., and 10:30 a.m. does not equal to the maximum number of sensors allowed under a budget constraint. This is because of two key reasons. (1) as the duration of operation increases, the associated cost of a sensor also increases, thereby fewer sensors can be deployed in the network. When duration of operation is 30 min, the corresponding heterogeneous sensor deployment strategy with the lowest variability has the maximum number of sensors, while the number of sensors is the minimum, when duration of operation is set as 120 min (equals to the analysis period). (2) the amount of information provided by each sensor also depends on its start time. The results show that the proposed algorithm can identify the optimal strategy by performing a tradeoff in terms of the number of each type of sensor and duration of operation. In addition, the results also illustrate the importance of incorporating sensor operation strategy in NSLP models. In summary, the optimal sensor deployment (the numbers of link and node sensors and their installation locations) and operation strategies (start time and duration of operation for which traffic data measurements are collected) can be determined using the proposed algorithm.

In summary, as shown in Table 1 and Figure 2, the proposed NSLP model and algorithm can capture the time-varying characteristics of the amount of information provided by each sensor location. The optimal sensor deployment and operation strategy in terms of the optimal combination of sensor types, the number, location and operation duration of each sensor can be determined, since this strategy can provide the largest amount of updated information to estimate dynamic O-D demand compared to any other possibilities.

**Figure 4:**
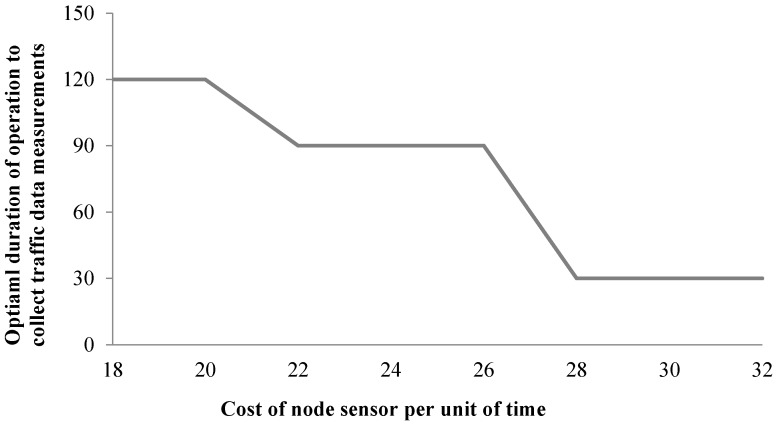
Optimal duration of operation for which traffic data measurements are collected with different node sensor cost per unit of time.

To study the impact of the operation cost of a sensor on the optimal heterogeneous sensor deployment and operation strategies, a numerical experiment was set up to demonstrate how the optimal duration of operation for which traffic data measurements are collected changes with the operation cost of a sensor per unit of time (i.e., $e_{v}$ and $e_{s}$ in Equations (27) and (28)). The start time is set to be 8:30 a.m., and only node sensors are considered for deployment. The maximum duration of operation is set to be 120 min, and the minimum duration of operation is set to be 30 min. As shown in Figure 4, the optimal duration of operation for each node sensor decreases when the cost of a node sensor per unit of time increases. When the operation cost of a node sensor per unit of time is low, the proposed algorithm tends to assign longer duration of operation to each sensor in favor of adding more sensors in the network for the optimal heterogeneous sensor deployment and operation strategies. As illustrated by Table 1, when the duration of operation increases for each node sensor, the allowed maximum number of sensors decreases as the operation cost of a sensor increases with the duration of operation. When the operation cost of a sensor per unit of time is low, the cost of each sensor does not increase significantly with the duration of operation. When duration of operation increases, the allowed maximum number of sensors does not decrease significantly. It means that when the cost of a sensor per unit of time is low, longer duration of operation with fewer sensors can provide a greater amount of updated information compared to shorter duration of operation with more sensors under a budget constraint. When the cost of a sensor per unit of time is high, the duration of operation in the optimal heterogeneous sensor deployment and operation strategies tends to be smaller due to budget constraints. In this case, the cost of each node sensor can increase significantly with the duration of operation increases. When the duration of operation increases, the allowed maximum number of sensors can decrease significantly. It means that when the cost of a sensor per unit of time is high, shorter duration of operation with more sensors can provide a greater amount of updated information compared to longer duration of operation with much fewer sensors.

## 5. Conclusions

This study proposes a network sensor location problem model to integrate the optimal heterogeneous sensor deployment (link and node sensors numbers and their installation locations) and operation (sensors’ start time and duration of operation for which traffic data measurements are collected) strategies for the dynamic O-D demand estimation problem under a budget constraint. The proposed model can be used to determine the optimal sensor deployment and operation strategies with the maximized quality or minimized variability of the O-D demand estimates. To measure the variability of the O-D demand estimates, the trace of the covariance matrix of the posterior O-D demand estimates is adopted. The proposed NSLP also factors the correlation between the operation cost of a sensor and its duration of operation, which has not been addressed in previous studies. An integrated sensor location and operation algorithm is developed to solve the proposed NSLP model under the multivariate normal distribution assumption for the prior O-D demand. 

To demonstrate the performance of the proposed NSLP model and its solution algorithm, numerical experiments are used. The results show that the proposed algorithm has the capability to identify the optimal sensor deployment and operation strategies with the lowest variability in the dynamic O-D demand estimates under a budge constraint. When the operation strategy is fixed, the results show that the variability of the dynamic O-D demand estimates decreases when the number of sensors increases, and the variability decreases more rapidly when a node sensor is added compared to a link sensor is added. This is because a node sensor can detect turning movements at intersections, and thus collects more information compared to a link sensor. However, under a budget constraint, more node sensors cannot guarantee the reduction of the variability of the dynamic O-D demand estimates. Because the cost of a link sensor is cheaper than the cost of a node sensor in our assumption, and more node sensors lead to fewer link sensors in the network. The results of computational time analysis show that the cumulative computational time to identify the sensor deployment strategy with a fixed operation strategy is approximately positively linearly correlated with the number of identified sensor locations, and the computational time to identify a sensor location with lower priority is smaller than the time to identify a sensor location with higher priority.

The results show that when the operation strategies change, the corresponding sensor deployment strategies with the lowest variability also change. The longer duration of operation for which traffic data measurements are collected by a sensor can provide a greater amount of updated information, which results a lower variability of the O-D demand estimates. Under a budget constraint, a longer duration of operation normally leads to fewer sensors being deployed, and vice versa. The proposed algorithm allows a tradeoff in terms of duration of operation and number of sensors under a budget constraint. The results also show that when the operation cost of a sensor per unit of time increases, the duration of operation in the optimal heterogeneous sensor deployment and operation strategies decreases. When the operation cost of a sensor per unit of time is high, the optimal heterogeneous sensor deployment and operation strategies tend to have more sensors with shorter duration of operation rather than fewer sensors with longer duration of operation. Hence, in dynamic O-D demand estimation, it is important to integrate heterogeneous sensor deployment and operation strategies. 

The proposed model and algorithm can be used to effectively design the integrated optimal heterogeneous sensor deployment and operation strategies, including sensor types, numbers, locations, start time, and duration of operation for which traffic data measurements are collected for dynamic O-D demand estimation under a budge constraint. A potential future research direction is to consider different cost functions adopted in practice to study the impact of the cost of sensors on the optimal heterogeneous sensor deployment and operation strategies. Another potential research direction is to enhance the quality of the dynamic O-D demand estimates by leveraging mobile sensors with sensors with fixed locations in the NSLP model. Furthermore, additional numerical experiments can be performed to evaluate the performance of the proposed model and algorithm in various larger transportation networks. Finally, additional studies are needed to develop various types of solution algorithms for the proposed model. Their effectiveness can be evaluated and compared to reduce the computation time in larger transportation networks for the fruition of the proposed model and algorithm adopted in practice.

## Figures and Tables

**Table 1:** Sensor Deployment Strategies with different start time and duration of operation for which traffic data measurements are collected.

Start Time (a.m.)	Duration of Operation (Minutes)	Sensor Deployment Strategy	Objective Function Value
**8:30**	30	7(n), 2(n), 14(n), 14–60, 10(n), 13(n), 16(n), 10–13, 14–16, 16–17	24,061.79
	**60**	**7(n), 2(n), 3(n), 3–2, 3–5, 2–3, 3–31**	**23,823.20**
	90	7(n), 3(n), 5–7, 5–3, 1–19	24,067.82
	120	7(n), 3(n), 1–19	23,945.14
9:00	30	7(n), 2(n), 14(n), 16(n), 11(n), 11–13, 19–18, 16–17, 1–19, 10–7, 14–13	23,905.52
	60	7(n), 3(n), 5(n), 14(n), 2–3, 5–7	23,919.72
	90	7(n), 3(n), 5–7, 5–3, 2–3	24,107.06
	120	7(n), 3(n), 5–7	24,295.98
9:30	30	7(n), 2(n), 3(n), 10(n), 13(n), 16(n), 7–10, 10–7, 13–14, 18–16	23,894.54
	60	7(n), 3(n), 10(n), 16(n), 19–47, 5–7	23,847.10
	90	7(n), 3(n), 3(n), 5–7, 16–17	24,287.76
	120	7(n), 3(n), 5–7	24,882.84
10:00	30	7(n), 2(n), 14(n), 3(n), 16(n), 10(n), 13(n), 13–14, 14–13	24,590.35
	60	7(n), 2(n), 14(n), 16(n), 1–44, 16–17	24,686.24
	90	7(n), 2(n), 16(n), 16–17	24,586.99
	120	7(n), 3(n), 64–7	25,275.38

## References

[1] Lu Z., Rao W., Wu Y., Guo L., Xia J. (2015). A Kalman filter approach to dynamic OD flow estimation for urban road networks using multi-sensor data. J. Adv. Transp..

[2] Caggiani L., Ottomanelli M., Sassanelli D. (2013). A Fixed Point Approach to Origin–Destination Matrices Estimation Using Uncertain Data and Fuzzy Programming on Congested Networks. Transp. Res. Part C.

[3] Lauren A., Jiang S., Murga M., González M.C. (2015). Origin–Destination Trips by Purpose and Time of Day Inferred from Mobile Phone Data. Transp. Res. Part C.

[4] Lam W.H.K., Lo H.P. (1990). Accuracy of O-D Estimates from Traffic Counting Stations. Traffic Eng. Control.

[5] Yang H., Iida Y., Sasaki T. (1991). An Analysis of the Reliability of an Origin-Destination Trip Matrix Estimated from Traffic Counts. Transp. Res. Part B.

[6] Yang H., Zhou J. (1998). Optimal Traffic Counting Locations for Origin-Destination Matrix Estimation. Transp. Res. Part B.

[7] Yim K.N., Lam W.H.K. (1998). Evaluation of Count Location Selection Methods for Estimation of OD Matrices. J. Transp. Eng..

[8] Bianco L., Confessore G., Reverberi P. (2001). A Network based Model for Traffic Sensor Location with Implications on O-D Matrix Estimates. Transp. Sci..

[9] Gan L., Yang H., Wong S.C. (2005). Traffic Counting Location and Error Bound in Origin-Destination Matrix Estimation Problems. J. Transp. Eng..

[10] Bierlaire M. (2002). The Total Demand Scale: A New Measure of Quality for Static and Dynamic Origin-Destination Trip Table. Transp. Res. Part B.

[11] Chen A., Chootinan P., Ryu S., Wong S.C. (2012). Quality Measures for Origin-Destination Estimation from Traffic Counts: A Review and a Generalized Demand Scale Measure. J. Transp. Eng..

[12] Simonelli F., Marzano V., Papola A., Vitiello I. (2012). A Network Sensor Location Procedure Accounting for O-D Matrix Estimate Variability. Transp. Res. Part B.

[13] Zhu S., Cheng L., Chu Z., Chen A., Chen J. (2014). Identification of Network Sensor Locations for Traffic Flow Estimation. Transp. Res. Rec..

[14] Chen A., Chootinan P., Pravinvongvuth S. (2004). Multiobjective Model for Locating Automatic Vehicle Identification Readers. Transp. Res. Rec..

[15] Chen A., Pravinvongvuth S., Chootinan P. (2010). Scenario-based Multiobjective AVI Reader Location Models under Different Travel Demand Patterns. Transportmetrica.

[16] Minguez R., Sanchez-Cambronero S., Castillo E., Jimenez P. (2010). Optimal Traffic Plate Scanning Location for OD Trip Matrix and Route Estimation in Road Networks. Transp. Res. Part B.

[17] Castillo E., Jiménez P., Menéndez J.M., Nogal M. (2013). A Bayesian Method for Estimating Traffic Flows based on Plate Scanning. Transportation.

[18] Yang H., Yang C., Gan L.P. (2006). Models and Algorithms for the Screen Line-based Traffic-Counting Location Problems. Comput. Oper. Res..

[19] Chen A., Pravinvongvuth S., Chootinan P., Lee M., Recker W. (2007). Strategies for Selecting Additional Traffic Counts for Improving O-D Trip Table Estimation. Transportmetrica.

[20] Hu S.-R., Peeta S., Liou H.-T. (2015). Integrated Determination of Network Origin-Destination Trip Matrix and Heterogeneous Sensor Selection and Location Strategy. IEEE Trans. Intell. Transp. Syst..

[21] Eisenman S.M., Fei X., Zhou X., Mahmassani H.S. (2006). Number and Location of Sensors for Real-Time Network Traffic Estimation and Prediction: Sensitivity Analysis. Transp. Res. Rec..

[22] Fei X., Mahmassani H.S., Eisenman S.M. Sensor Coverage and Location for Real-Time Traffic Prediction in Large-Scale Networks. Presented at the 86th Annual Meeting of the Transportation Research Board.

[23] Asakura Y., Hato E., Kashiwadani M. (2000). Origin-Destination Matrices Estimation Model Using Automatic Vehicle Identification Data and its Application to the Han-Shin Expressway Network. Transportation.

[24] Zhou X., List G.F. (2010). An Information-Theoretic Sensor Location Model for Traffic Origin-Destination Demand Estimation Applications. Transp. Sci..

[25] Barcelo J., Gillieron F., Linares M., Serch O., Montero L. (2012). Exploring link covering and node covering formulations of detection layout problem. Transp. Res. Rec..

[26] Zhu S., Hong Z., Peeta S., Guo Y., Cheng L., Sun W. (2016). Optimal Heterogeneous Sensor Deployment Strategy for Dynamic Origin-Destination Demand Estimation. Transp. Res. Rec..

[27] Viti F., Verbeke W., Tampère C.M.J. (2008). Sensor Locations for Reliable Travel Time Prediction and Dynamic Management of Traffic Networks. Transp. Res. Rec..

[28] Xing T., Zhou X., Taylor J. (2013). Designing Heterogeneous Sensor Networks for Estimating and Predicting Path Travel Time Dynamics: An Information-Theoretic Modeling Approach. Transp. Res. Part B.

[29] Evangelos M., Evangelia C., Josep M.S.G., Panagiotis I., Georgia A. (2017). The sensor location problem: Methodological approach and application. Transport.

[30] Bianco L., Cerrone C., Cerulli R., Gentili M. (2014). Locating Sensors to Observe Network Arc Flows: Exact and Heuristic Approaches. Comput. Oper. Res..

[31] Castillo E., Jiménez P., Menéndez J.M., Conejo A.J. (2008). The Observability Problem in Traffic Models: Algebraic and Topological Methods. IEEE Trans. Intell. Transp. Syst..

[32] Miralinaghi M., Lou Y., Keskin B.B., Zarrinmehr A., Shabanpour R. (2014). Refueling Station Location Problem with Traffic Deviation Considering Route Choice and Demand Uncertainty. Int. J. Hydrogen. Energy.

[33] Miralinaghi M., Lou Y., Hsu Y. (2016). Multiclass Fuzzy User Equilibrium with Endogenous Membership Functions and Risk-Taking Behavior. J. Adv. Transp..

[34] Ng M. (2012). Synergistic Sensor Location for Link Flow Inference without Path Enumeration: A Node-based Approach. Transp. Res. Part B.

[35] He S. (2013). A Graphical Approach to Identify Sensor Locations for Link Flow Inference. Transp. Res. Part B.

[36] Xu X., Hong K.L., Chen A., Castillo E. (2016). Robust network sensor location for complete link flow observability under uncertainty. Transp. Res. Part B.

[37] Gentili M., Mirchandani P. (2011). Survey of models to locate sensors to estimate traffic flows. Transp. Res. Rec..

[38] Gentili M., Mirchandani P. (2005). Locating active sensors on traffic networks. Ann. Oper. Res..

[39] Guo Y., He X., Peeta S., Weiss J. (2016). Internal Curing for Concrete Bridge Decks: Integration of a Social Cost Analysis in Evaluation of Long-Term Benefit. Transp. Res. Rec..

[40] Lam W.H.K., Shao H., Sumalee A. (2008). Modeling Impacts of Adverse Weather Conditions on a Road Network with Uncertainties in Demand and Supply. Transp. Res. Part B.

[41] Shao H., Lam W.H.K., Sumalee A., Chen A., Hazelton M.L. (2014). Estimation of Mean and Covariance of Peak Hour Origin–Destination Demands from Day-to-Day Traffic Counts. Transp. Res. Part B.

[42] Maher M.J. (1983). Inferences on Trip Matrices from Observations on Link Volumes: A Bayesian Statistical Approach. Transp. Res. Part B.

[43] Castillo E., Menéndez J.M., Sánchez-Cambronero S. (2008). Predicting Traffic Flow Using Bayesian Networks. Transp. Res. Part B.

